# Measurement tools of resource use and quality of life in clinical trials for dementia or cognitive impairment interventions: protocol for a scoping review

**DOI:** 10.1186/s13643-017-0418-6

**Published:** 2017-01-26

**Authors:** Fan Yang, Piers Dawes, Iracema Leroi, Brenda Gannon

**Affiliations:** 1 Manchester Centre for Health Economics, The University of Manchester, Manchester, UK; 2 Manchester Centre for Audiology and Deafness, The University of Manchester, Manchester, UK; 3 Institute of Brain, Behaviour and Mental Health, The University of Manchester, Manchester, UK; 4 The University of Queensland, Centre for Business and Economics of Health, Brisbane, Australia

**Keywords:** Resource use, Quality of life, Tool, Clinical trial, Dementia, Cognitive impairment

## Abstract

**Background:**

Dementia and cognitive impairment could severely impact patients’ life and bring heavy burden to patients, caregivers and societies. Some interventions are suggested for the older patients with these conditions to help them live well, but economic evaluation is needed to assess the cost-effectiveness of these interventions. Trial-based economic evaluation is an ideal method; however, little is known about the tools used to collect data of resource use and quality of life alongside the trials. Therefore, the aim of this review is to identify and describe the resource use and quality of life instruments in clinical trials of interventions for older patients with dementia or cognitive impairment.

**Methods:**

We will perform a search in main electronic databases (Ovid MEDLINE, PsycINFO, EMBASE, CINAHL, Cochrane Databases of Systematic Reviews, Web of Science and Scopus) using the key terms or their synonyms: older, dementia, cognitive impairment, cost, quality of life, intervention and tools. After removing duplicates, two independent reviewers will screen each entry for eligibility, initially by title and abstract, then by full-text. A hand search of the references of included articles and general search, e.g. Google Scholar, will also be conducted to identify potential relevant studies. All disagreements will be resolved by discussion or consultation with a third reviewer if necessary. Data analysis will be completed and reported in a narrative review.

**Discussion:**

This review will identify the instruments used in clinical trials to collect resource use and quality of life data for dementia or cognitive impairment interventions. This will help to guide the study design of future trial-based economic evaluation of these interventions.

**Systematic review registration:**

PROSPERO CRD42016038495

**Electronic supplementary material:**

The online version of this article (doi:10.1186/s13643-017-0418-6) contains supplementary material, which is available to authorized users.

## Introduction

As the population ages rapidly, the prevalence of dementia and cognitive impairment is a growing public health concern worldwide and it has been estimated to increase within the next 20 years [1]. These two disorders could impact patients’ cognitive function, behaviour and activities of daily living, and have become one of the principal causes of disability and decreased quality of life (QoL) among older people [2]. It is increasingly recognised that psychosocial interventions contribute to the care of people with dementia and their families in a wide range of domains [3]. For example, the sensory rehabilitation has been shown to improve patients’ QoL and increase their social engagement, which could help them live well with dementing conditions [4].

In light of expanding health care costs and finite budget, cost-effectiveness analysis is essential for national health care decisions and resource allocation. The outcome of effectiveness used in such analysis is the quality-adjust life years (QALYs), which take both the quantity and quality of life into account. In dementia research, QoL has been recognised as an important measure as the clinical efficacy measure [5]. Several instruments have specifically been developed to assess QoL in dementia [2, 6, 7]. According to the most recent systematic review [6], more than 10 QoL measures were identified and properties assessed, but this review was limited to disease-specific QoL measures only, and such measures may not be used directly to generate health utility scores for QALYs calculations in cost-effectiveness analysis.

Among the methods available to assess health care interventions, trial-based economic evaluations are considered as an ideal vehicle for data generation because of the availability of patient-level data and unbiased estimates of clinical outcomes [8]. But more information is needed on the tools for data collection alongside the trials. Schölzel-Dorenbos et al. [2] performed a systematic review in 2006 on the use of QoL measures as an outcome in intervention trials in patients with mild cognitive impairment or dementia and found only three studies and two QoL scales. Following this review, a lot of new QoL instruments were developed and widely used, e.g., Dementia Quality of Life questionnaire (DEMQOL) [9] and dementia specific quality of life instrument (QUALIDEM) [10].

Resource use is also an essential component in the cost-effectiveness analysis. Instruments are recommended for cost data collection to improve the quality and uniformity of data generated from trials, suggested by the International Society for Pharmacoeconomics and Outcomes Research (ISPOR) [11]. The Resource Utilisation in Dementia (RUD) instrument is a standardised tool and the most widely used instrument for resource use data collection in dementia [12]. It has been used in several clinical drug trials for Alzheimer’s disease [13–15] and several observational studies [16–18]. But there is a lack of information about the use of RUD in clinical trials for dementia or cognitive impairment, especially for non-pharmacological interventions, and whether there are other instruments available to collect resource use data in such trials is yet unknown.

Therefore, in this review, we aim to identify and describe the resource use and QoL instruments that have been used in clinical trials of dementia or cognitive impairment interventions.

## Methods

This review will follow the Preferred Reporting Items for Systematic Reviews and Meta-Analysis (PRISMA) Statement [19] and consists of acquiring, extracting and assessing the data. This protocol is in accordance with the PRISMA-Protocols (PRISMA-P) 2015 checklist [20] (Additional file 1: Table S1 for the PRISMA-P 2015 checklist)

### Eligibility criteria

Studies fulfilling the following criteria will be included in the systematic review:

- Population—older adults with dementia or cognitive impairment
- Intervention—all types of interventions, both drug and nondrug therapies
- Comparator—no intervention or the usual care
- Outcomes—measurement and reporting of QoL, or resource use or both
- Study type—randomised clinical trial, or feasibility study or pilot study

No language restrictions will be imposed during the literature search but the abstract should be available in English. There is no restriction on date of publication. All studies should be original research published in a peer-reviewed journal. For the definition of ‘older adults’ used in this review, we will accept any age cut-off if a study describes their population as being ‘older adults’. The definition of ‘patients with dementia or cognitive impairment’ will also be based on each individual study. The outcomes should be measured using standardised questionnaires or tools. Quality of life is an abstract and broad concept including physical function, perceptions of well-being, satisfaction, and sense of self-worth. Given the aim to guide cost-effectiveness analysis study design, quality of life, quality-adjusted life years, health utility and QALY will be used as the search terms.

### Information sources

The following major databases for the discipline of medicine and nursing will be searched: Ovid MEDLINE, PsycINFO, EMBASE, CINAHL, Cochrane Databases of Systematic Reviews, Web of Science and Scopus. The systematic search will be conducted in September 2016, and the searches will be re-run just before the final analyses to retrieve further studies for inclusion. A hand search of the references of included articles and general search, e.g. Google Scholar, will also be conducted to identify potential relevant studies.

### Search strategy

Key terms have been determined through discussion between two authors (FY and BG). The following terms or their synonyms will be used: older, dementia, cognitive impairment, cost, quality of life, intervention and tools. The search terms used in Ovid MEDLINE can be found in Additional file 2: Table S2. The search strategies will be created specifically for each database using relevant index and free text terms. The titles and abstracts of all identified studies potentially eligible for inclusion in the review will be screened. Full-text versions of the included articles will be obtained.

### Data management

All results from database and hand searches will be exported into Endnote X7 software (Thomson Reuters, 2016). Duplicates will be removed using a standard function before each entry will be screened from eligibility. After dropping duplicates, all the titles and abstracts of the studies retrieved will be imported to an Excel spreadsheet (Microsoft Corporation, 2010) for screening.

### Study selection

Study selection will be undertaken in two stages: first, titles and abstracts will be screened against the inclusion criteria; second, the full-text for all eligible articles will be screened to confirm whether or not the study should be included in the final review. Two authors (FY and BG) will carry out the selection process. If there are discrepancies and the two investigators cannot reach a consensus, the disagreements will be resolved through discussion and consultation with a third reviewer (PD).

### Data extraction

One review author (FY) will extract data using a standardised data extraction form developed for this review (Additional file 3: Table S3). A second author (BG) will then verify the extracted data. Any discrepancies will be resolved through discussion or adjudication by a third author (PD), until consensus is reached. The data extracted for this review will include:

1. Publication characteristics (title, year of publication, author, study objective, type of study);
2. Participant characteristics (country, inclusion criteria, exclusion criteria, age, sex, and disease, e.g. mild cognitive impairment, dementia, or both);
3. Intervention characteristics (what intervention, type of intervention, duration of intervention and comparator);
4. Outcome characteristics (whether a cost/QoL measure was used, what instrument or instruments used, time points at which the instrument was assessed, patient/proxy reported, and type of QoL measure).

If any of the previously described data is not clearly presented in the research article, we will contact the authors for clarification by sending emails.

### Quality assessment

Since the aim of this review is to identify and describe measures of resource use and QoL in trials, without reporting quantitative evaluation of measurement properties or trial effect estimates, the quality of included studies will not be assessed.

### Data synthesis

First, the characteristics of included studies will be tabulated based on the data extracted using Additional file 3: Table S3. Second, the frequency of each resource use or QoL instrument used in the trials will be reported. Third, the characteristic of each measurement instrument will be summarised using a table (Additional file 4: Table S4), which is based on the summary table used in one systematic review of dementia-specific QoL scales [6], and will include instrument name, conceptual basis, patient report (Yes/No), proxy report (Yes/No), patient population, subscales, items, response options and scoring. One author (FY) will summarise the results, and a second researcher (BG) will review and highlight any discrepancies. Disagreements between the two authors will be resolved by discussion, with involvement of a third review author (PD) where necessary.

## Discussion

The main aim of this review is to identify and describe the tools/instruments used in clinical trials to collect data of resource use and QoL for dementia or cognitive impairment interventions. The results of the review will provide information about potentially useful instruments in future similar trials and contribute to the study design of trial-based economic evaluation of dementia or cognitive impairment interventions. We anticipate that the review will be useful to a variety of stakeholders who have an interest in dementia and cognitive impairment care.

## Additional files

Additional file 1: Table S1. PRISMA-P 2015 checklist. (DOC 82 kb)
Additional file 2: Table S2. Search terms for Ovid MEDLINE. (DOCX 17 kb)
Additional file 3: Table S3. Data extraction form. (DOC 42 kb)
Additional file 4: Table S4. Characteristics of resource and QoL measures. (DOC 28 kb)
