# Supplementary material for: Measurement tools of resource use and quality of life in clinical trials for dementia or cognitive impairment interventions: protocol for a scoping review
Source: Syst Rev. 2017 Jan 26;6:22. doi: 10.1186/s13643-017-0418-6 (PMC5270230; doi:10.1186/s13643-017-0418-6)
Supplement: Additional file 2: Table S2. — Search terms for Ovid MEDLINE. (DOCX 17 kb) [file 13643_2017_418_MOESM2_ESM.docx]

**Table S2. Search terms for Ovid Medline**

| 1 | exp dementia/ or exp alzheimer disease/ or exp cognitive impairment/ |
| --- | --- |
| 2 | sensory impairment.ti,ab. |
| 3 | 1 or 2 |
| 4 | exp aged/ or exp frail elderly/ |
| 5 | (older or old or elderly or senior or "over 65" or "65 year*").ti,ab. |
| 6 | 4 or 5 |
| 7 | Economics, Medical/ or Economics/ or Economics, Hospital/ or Economics, Nursing/ |
| 8 | exp "Costs and Cost Analysis"/ |
| 9 | Health Expenditures/ |
| 10 | ("resource use" or "health care utilisation" or "health care utilization" or "resource utilisation" or "resource utilization").ti,ab. |
| 11 | 7 or 8 or 9 or 10 |
| 12 | "Quality of Life"/ |
| 13 | Quality-Adjusted Life Years/ |
| 14 | ("health utility" or QALY).ti,ab. |
| 15 | 12 or 13 or 14 |
| 16 | 11 or 15 |
| 17 | (intervention or program or promotion).ti,ab. |
| 18 | Clinical Trial/ |
| 19 | Feasibility Studies/ |
| 20 | Pilot Projects/ |
| 21 | (trial or feasibility or pilot).ti,ab. |
| 22 | 18 or 19 or 20 or 21 |
| 23 | 3 and 6 and 16 and 17 and 22 |
|  |  |
