# Supplementary material for: Measurement tools of resource use and quality of life in clinical trials for dementia or cognitive impairment interventions: protocol for a scoping review
Source: Syst Rev. 2017 Jan 26;6:22. doi: 10.1186/s13643-017-0418-6 (PMC5270230; doi:10.1186/s13643-017-0418-6)
Supplement: Additional file 3: Table S3. — Data extraction form. (DOC 42 kb) [file 13643_2017_418_MOESM3_ESM.doc]

**Table S3. Data extraction form**

| **Data to be extracted** | **Item** | **Notes to the reviewer** |
| --- | --- | --- |
| **Publication characteristics** | Title |  |
|  | Year of publication |  |
|  | Author |  |
|  | Study objective |  |
|  | Type of study (feasibility study/pilot study/RCT) |  |
| **Participant characteristics** | Country |  |
|  | Inclusion criteria |  |
|  | Exclusion criteria |  |
|  | Age |  |
|  | Sex |  |
|  | Disease (mild cognitive impairment/dementia/both/others) |  |
| **Intervention characteristics** | What intervention(s)? |  |
|  | Type of interventions (drug/non-drug) |  |
|  | Duration of interventions |  |
|  | Comparator |  |
| **Outcome characteristics** | Cost measure used (Yes/No) |  |
|  | What cost measure? |  |
|  | Cost measure time point |  |
|  | Patient/Proxy reported cost measure? |  |
|  | QoL measure used (Yes/No) |  |
|  | What QoL measure? |  |
|  | QoL time point |  |
|  | Patient/Proxy reported QoL measure? |  |
|  | Type of QoL measure (disease-specific/generic) |  |
|  | Type of QoL measure (profiled-based/preference-based) |  |
| **Overall conclusion** |  |  |
