# Supplementary material for: Measurement tools of resource use and quality of life in clinical trials for dementia or cognitive impairment interventions: protocol for a scoping review
Source: Syst Rev. 2017 Jan 26;6:22. doi: 10.1186/s13643-017-0418-6 (PMC5270230; doi:10.1186/s13643-017-0418-6)
Supplement: Additional file 4: Table S4. — Characteristics of resource and QoL measures. (DOC 28 kb) [file 13643_2017_418_MOESM4_ESM.doc]

**Table S4. Characteristics of resource use and QoL measures**

| Instrument | Conceptual basis | Patient report  (Yes/No) | Proxy report  (Yes/No) | Patient population | Subscales | Items | Response options | Scoring |
| --- | --- | --- | --- | --- | --- | --- | --- | --- |
|  |  |  |  |  |  |  |  |  |
|  |  |  |  |  |  |  |  |  |
|  |  |  |  |  |  |  |  |  |
|  |  |  |  |  |  |  |  |  |
